# Therapeutic Efficacy of Alpha-Lipoic Acid against Acute Myocardial Infarction and Chronic Left Ventricular Remodeling in Mice

**DOI:** 10.1155/2020/6759808

**Published:** 2020-01-07

**Authors:** Zequan Yang, Yikui Tian, Stuart S. Berr, Brent A. French

**Affiliations:** ^1^Department of Surgery, University of Virginia Health System, Charlottesville, VA 22908, USA; ^2^Department of Biomedical Engineering, University of Virginia Health System, Charlottesville, VA 22908, USA; ^3^Department of Radiology, University of Virginia Health System, Charlottesville, VA 22908, USA; ^4^Department of Cardiovascular Medicine, University of Virginia Health System, Charlottesville, VA 22908, USA

## Abstract

**Background:**

We hypothesized that daily administration of a potent antioxidant (*α*-lipoic acid: ALA) would protect the heart against both acute myocardial infarction (AMI) and left ventricular remodeling (LVR) post-AMI.

**Methods and Results:**

Two separate studies were conducted. In the AMI study, C57Bl/6 mice were fed ALA daily for 7 d prior to a 45-minute occlusion of the left coronary artery (LCA). Mean infarct size in control mice (fed water) was 60 ± 2%. Mean infarct size in ALA-treated mice was 42 ± 3% in the 15 mg/kg·d group and 39 ± 3% in the 75 mg/kg·d group (both *P* < 0.05 vs. control). In the LVR study, AMI increased LV end-systolic volume (LVESV) and reduced LV ejection fraction (LVEF) to a similar extent in both groups when assessed by cardiac MRI 1 day after a 2-hour LCA occlusion. Treatment with ALA (75 mg/kg·d) or H_2_O was initiated 1 day post-AMI and continued until study's end. Both LVESV and LVEF in ALA-treated mice were significantly improved over control when assessed 28 or 56 days post-AMI. Furthermore, the survival rate in ALA-treated mice was 63% better than in control mice by 56 days post-AMI.

**Conclusions:**

Daily oral ingestion of ALA not only protects mice against AMI but also attenuates LVR and preserves contractile function in the months that follow.

## 1. Introduction

It has long been known that reactive oxygen species (ROS) play a key role in the pathophysiology of acute myocardial infarction (AMI) [1–3]. Myocardial ischemia/reperfusion injury is also associated with reduced cellular levels of endogenous antioxidants and antioxidant enzymes, particularly in the chronic setting post-AMI [2, 4]. Recent studies indicate that oxidative stress persists in the myocardium after AMI [4–8] and secondary prevention trials of antioxidant vitamins such as vitamin E and *β*-carotene have yielded encouraging results [9]. However, these same clinical trials failed to demonstrate a reduction in the risk of fatal coronary events, perhaps because the antioxidant vitamins under investigation were intrinsically weak and/or provided a limited spectrum of antioxidant protection. Like vitamin E and *β*-carotene, alpha-lipoic acid (ALA) is a naturally-occurring antioxidant that is widely available as a nutritional supplement. Unlike vitamin E and *β*-carotene, ALA is a relatively potent antioxidant with a broad spectrum of antioxidant properties [10] that may make it more efficacious in disease states where oxidative stress contributes to cardiovascular pathophysiology [11–13].

ALA is a coenzyme of the pyruvate and alpha-ketoglutarate dehydrogenase enzyme complexes. Upon cellular uptake, ALA is rapidly converted to its reduced form: dihydrolipoic acid (DHLA). The presence of two reduced thiol groups in DHLA makes it a potent antioxidant. Thiol-based antioxidants serve critical functions in maintaining redox homeostasis [12]. Not only do they protect lipid membranes, proteins and DNA against oxidative damage imposed by reactive oxygen species (ROS), but redox-based reactions are now known to play critical roles in intracellular signaling and gene expression regulation [14].

ALA and DHLA are known to enhance cellular antioxidant capacity and react with multiple ROS [10]. ALA is capable of increasing intracellular glutathione and coenzyme Q_10_ levels [15]. These properties make ALA an appealing probe for evaluating the role of ROS in the pathophysiology of cardiovascular disease.

The protective effects of ALA have previously been studied in animal models of cerebral ischemia/reperfusion injury [16]. ALA also reduces infarct size and improves functional recovery in rat myocardial ischemia/reperfusion injury [11, 17]. However, the potential of ALA to limit the progression of LVR after AMI has not previously been examined in intact animals.

In the present study, the cardioprotective potential of ALA was examined *in vivo* using two separate mouse models. Cardioprotection in the setting of AMI was tested by treating mice with ALA for 1 week prior to a reperfused coronary occlusion. ALA was tested in the postinfarction setting by subjecting mice to reperfused AMI, then initiating daily treatment with ALA for the duration of the study. The results demonstrate that mice pretreated with ALA are more resistant to AMI, and that ALA treatment (even when initiated post-AMI) helps preserve both cardiac structure and function.

## 2. Materials and Methods

### 2.1. Experimental Myocardial Infarction

These studies conform to the “Guide for the Care and Use of Laboratory Animals” (NIH publication, 8^th^ edition, revised in 2011) and were conducted under approved protocols using C57Bl/6 mice obtained from a licensed vendor (Jackson Laboratory, Bar Harbor, ME). AMI was induced by brief LCA occlusion followed by reperfusion as reported previously [3, 18]. In brief, mice were anesthetized with sodium pentobarbital (80 mg/kg i.p.) and orally intubated. Artificial respiration was maintained with a FiO_2_ of 0.80, 120 strokes per minute, and a 0.2 to 0.3 mL stroke volume. The heart was exposed through a left thoracotomy. A 7-0 silk was passed beneath the major left coronary artery (LCA) at the level of the lower left atrium. AMI was induced by ligating the suture over a piece of PE-20 tubing. Successful occlusions were verified by blanching in the area-at-risk and by changes in the ECG (widening of the QRS and ST segment elevation). Body temperature was maintained between 36.5 and 37.5°C using a rectal probe, digital thermometer, and heating lamp.

### 2.2. Drug Preparation and Administration

Alpha-lipoic acid (racemic mixture of DL-6,8-thioctic acid, Sigma Chemical Co., St. Louis, MO) was dissolved in water by adjusting the pH to 7.6 with dilute NaOH. The drug (or equal volume of vehicle) was administered daily by gavage using a 22G feeding tube at a volume of 2 *μ*l/g body weight per day for 7 days prior to AMI or for 56 days after AMI.

### 2.3. Acute Myocardial Infarction

#### 2.3.1. Mouse Model

After 7 days of daily ALA or vehicle treatment, AMI was induced by occluding the LCA for 45 min with a silk ligature. Twenty-four hours after reperfusion, mice were reanesthetized and ventilated in order to collect blood and remove the heart for infarct analysis.

#### 2.3.2. Experimental Groups

A total of 44 male mice (10–12 weeks old) were distributed into three groups. Group I was composed of control animals (*n* = 15) treated with water (2 *μ*l/g·day) for 7 days prior to AMI. Group II was composed of mice (*n* = 15) treated with ALA at 15 mg/kg·day and Group III of mice (*n* = 14) treated with ALA at 75 mg/kg·day for 7 days prior to AMI. Three mice from each group were euthanized 2 hours after reperfusion for blood samples and heart tissues. All other animals in the AMI study were euthanized for infarct analysis 24 hours after reperfusion. Blood samples were collected by puncturing the right ventricle, and the hearts were harvested for measurement of infarct size in mice with 24-hour reperfusion. The plasma was obtained after centrifuging the blood at 500 g for 8 minutes.

#### 2.3.3. Determination of Infarct Size

After excision, each heart was perfused via the ascending aorta with 2-3 ml of 37°C saline followed by 3-4 ml of 37°C 1.0% TTC in phosphate-buffered saline. After TTC staining, the LCA was reoccluded by tightening the silk suture that was intentionally left in the myocardium during surgery 24 hours earlier. The hearts were then perfused with 2 to 3 ml of 10% phthalo blue dye to delineate nonischemic tissue, and the left ventricle was cut into 5–7 transverse slices prior to fixation in 10% neutral-buffered formalin. Each slice was weighed and photographed. Digital images were then transferred to PhotoShop (Adobe) for analysis. The borders of the infarct area, area-at-risk for infarction, and nonischemic area were traced on both sides of each tissue slice to determine the corresponding areas as a function of pixel count. The weights of the nonischemic area, the area-at-risk, and the infarct area for each slice was calculated as a percentage of total pixel area multiplied by the total weight of the individual slice [3, 18].

### 2.4. Lipid Peroxidation Assay

Lipid peroxidation in plasma was measured in 2-hour and 24-hour reperfusion samples using a colorimetric assay (Calbiochem, La Jolla, CA) for malondialdehyde (MDA) [3]. Condensation of one molecule of MDA with 2 molecules of N-methyl-2-phenylindole yielded a stable chromophore with maximal absorbance at 586 nm. The concentration of MDA in each sample was calculated from a standard curve generated using authentic MDA (Sigma, St. Louis, MO).

### 2.5. Nitrate/Nitrite Assay

Nitrate/nitrite levels in the plasma were measured in samples collected at 2-hour postreperfusion. Plasma nitrate was first reduced to nitrite by incubation of 50 *μ*l plasma with 25 *μ*l nitrate reductase (600 mU/ml) and 25 *μ*l NADPH (160 *μ*mol/l) at 37°C for 90 min. Nitrite level was then assayed using a modified Griess reagent (Szechrome NIT; Polyscience, Warrington, PA) according to the manufacturer's instructions. In brief, plasma samples and sodium nitrite standards were incubated with 50 *μ*l of NIT reagent for 10 min. Absorbance was read at 542 nm, and plasma nitrate/nitrite levels were expressed in micromoles per milliliter.

### 2.6. Myeloperoxidase Activity Assay

After the heart was harvested at the end of 2-hour reperfusion, the right ventricle and atria were removed. The LV was separated into 3 pieces: ischemic (there was a clear-cut border between previously ischemic and nonischemic remote areas), adjacent (1 to 1.5 mm thickness of peri-ischemic region), and remote regions. Each piece was homogenized in a solution containing 0.5% hexadecyltrimethylammonium bromide dissolved in 10 mmol/L potassium phosphate buffer (pH 7) and centrifuged for 30 minutes at 20,000 g at 4°C. An aliquot of the supernatant was allowed to react with a solution of tetramethylbenzidine (1.6 mmol/L) and 0.1 mmol/L H_2_O_2_. The rate of the change in absorbance was measured with a spectrophotometer at 650 nm.

### 2.7. Left Ventricular Remodeling Study

#### 2.7.1. Mouse Model

Acute myocardial infarction (AMI) was induced by occluding the LCA for 2 hours. Twenty-four hours later, daily oral administration of ALA (or vehicle) was initiated and continued until the end of the 2-month study.

#### 2.7.2. Experimental Groups

Twenty-nine male mice (8–10 weeks old) were divided into three groups. Group IV was composed of control animals (*n* = 14) fed water (2 *μ*l/g·day) for 2 months after AMI. Group V was composed of mice (*n* = 10) fed ALA at a dose 75 mg/kg·day for 2 months after AMI. An additional group of mice (*n* = 5) was used to determine the infarct size that resulted from 2 hours of LCA occlusion followed by 24 hours of reperfusion (Group VI).

#### 2.7.3. Cardiac MRI

Mice were anesthetized with pentobarbital (60 mg/kg, IP) for cardiac MR imaging using a Helmholtz RF coil on a Varian Inova 4.7T MR scanner as reported previously [18]. An ECG-triggered, 2D cine FLASH sequence was employed with a slice thickness of 1 mm and an inplane resolution of 0.1 × 0.1 mm^2^. The flip angle was 20°, and the TR was 12–20 ms. During each imaging session, the entire LV was assessed using 6–9 contiguous short-axis slices. The images were scaled and converted to the appropriate format for analysis using the ARGUS software package (Siemens Medical Systems, Iselin, NJ). After semi-automated tracing of the endocardial borders, the end-diastolic and end-systolic phases were determined, and the left ventricular end-systolic volume (LVESV), the LV end-diastolic volume (LVEDV) and LV ejection fraction (LVEF) were automatically computed.

#### 2.7.4. Plasma MDA and Postmortem Determination of Infarct Extent

At the completion of the 2-month study, mice were euthanized, and blood and hearts were harvested. Plasma MDA was measured as described above. The hearts were perfused with 2-3 ml of 37°C saline followed by 3-4 ml of 37°C 1.0% TTC in phosphate-buffered saline. Hearts were frozen and cut into 1.0–1.5 mm thick transverse slices for fixation in 10% neutral-buffered formalin. The weights and thicknesses of each slice were measured before photographing both sides as described above. The methods used to calculate infarct extent [16] 2 months after AMI were different from the methods used to calculate infarct size 24 hours after AMI. For infarct extent, the LV midwall circumference and the fraction thereof containing scar tissue were first determined for each slice. These values were then multiplied by the individual slice thickness to obtain the extent of scar tissue in each slice. Infarct extent was then expressed as the sum of the values obtained from each of the slices.

### 2.8. Statistical Analysis

Data were reported as mean ± SEM. Infarct sizes and areas-at-risk in the AMI study and cardiac function and structure in LVR study were analyzed with one-way ANOVA followed by Student's *t*-tests for unpaired data with the Bonferroni correction. Survival data were analyzed using the Kaplan–Meier method.

## 3. Results

### 3.1. Mortality and Exclusions

The surgery-related mortality (defined as death during the procedure or within the first 24 hours after reperfusion) in the 73 mice undergoing surgery was 4%. In the AMI study, two mice (one each from the control and experimental groups) died overnight after coronary occlusion. Technical difficulties in determining the area-at-risk precluded the calculation of infarct size in 3 mice. In the LVR study, one mouse was excluded because it died of abdominal bleeding on day 1 post-AMI as a result of the injecting anesthetic prior to MRI.

### 3.2. Effect of ALA on AMI

#### 3.2.1. Procedural data

Both the ALA and vehicle treatments were well tolerated, with no morbidity or mortality noted as an immediate result of gavage administration. Neither significant changes in body weight were observed during the first week of treatment, nor were there any significant differences in heart rate during surgery among the three groups. Twenty-four hours after reperfusion, the ratio of LV weight to body weight was significantly higher in the control group than in the ALA-treated groups (0.361 ± 0.008% vs. 0.325 ± 0.010% & 0.337 ± 0.007%, *P* < 0.05), suggesting that myocardial edema was more severe in control mice. While increased LV-to-body weight ratio is commonly associated with cardiac hypertrophy, myocardial edema is more likely in this setting 24 hours post-AMI, consistent with previous studies employing T2-weighted MRI [19].

#### 3.2.2. Infarct Size

**Figure 1:**
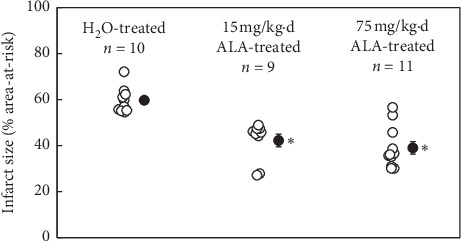
*α*-Lipoic acid (ALA) was administered daily for 7 d prior to a reperfused, 45-minute coronary occlusion. Two doses were examined: 15 and 75 mg/kg·day. Mean infarct size (% risk region) in ALA-treated mice was reduced by a minimum of 30% relative to control mice. ^*∗*^*P* < 0.05 as compared with the H_2_O-treated group.

There were no significant differences in the area-at-risk among the three groups. The mean infarct size (expressed as a percentage of the area-at-risk) in control mice was 60 ± 2%. Mean infarct size in ALA-treated mice was 42 ± 3% in the 15 mg/kg·day group (*n* = 9) and 39 ± 3% in the 75 mg/kg·day group (*n* = 11) (both *P* < 0.05 vs. control) (Figure 1). No significant difference in infarct size was found between the two groups treated with different doses of ALA.

#### 3.2.3. Lipid Peroxidation and Plasma Nitrate/Nitrite after AMI

**Figure 2:**
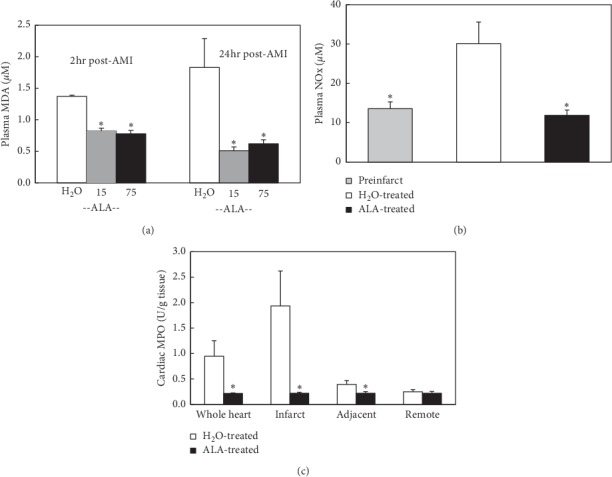
(a) Plasma malondialdehyde (MDA) levels were assessed 2 h and 24 h post-AMI. At 2 h post-AMI, MDA levels in either treatment group (15 or 75 mg/kg·day ALA) were reduced by over 40% relative to control (*P* < 0.05, either comparison). Twenty-four hours after AMI, MDA levels in either treatment group were reduced by nearly 70% relative to control (*P* < 0.05, either comparison). (b) Total plasma nitrate/nitrite 24 hours post-AMI was also reduced to control levels after 7-day pretreatment with 75 mg/kg·day ALA. (c) Myeloperoxidase activity in the infarct zone 24 hours post-AMI was reduced to control levels by 7-day pretreatment with 75 mg/kg·day ALA. ^*∗*^*P* < 0.05 as compared with the H_2_O-treated group.

When assessed 2 hours after reperfusion, plasma MDA levels in either the 15 and 75 mg/kg·day groups of ALA-treated mice were reduced by over 40% relative to control (*P* < 0.05, either comparison, Figure 2(a)). This difference was even more pronounced 24 hours after AMI, when MDA levels in both groups of ALA-treated mice were reduced by nearly 70% relative to control (*P* < 0.05, either comparison, Figure 2(a)). Similarly, plasma nitrate/nitrite was also significantly increased in control mice but significantly reduced in high-dose ALA-treated mice 2 hours after AMI (Figure 2(b)).

#### 3.2.4. Myocardial Tissue Myeloperoxidase Activity after AMI

Myocardial tissue myeloperoxidase activity was measured, respectively, in nonischemic remote area, adjacent to ischemic area and ischemic area 2 hours after AMI. The myeloperoxidase activity was significantly increased in the ischemic and adjacent areas in control mice. The activity was significantly reduced in high-dose ALA-treated mice (Figure 2(c)).

### 3.3. Effect of ALA on Left Ventricular Remodeling after AMI

#### 3.3.1. Infarct Size after 2-Hour Occlusion and 24-Hour Reperfusion

In a parallel study, five mice underwent 2 hours of LCA occlusion and 24 hours of reperfusion (Group VI) to define the resulting infarct size. The area-at-risk comprised 38 ± 3% of the LV mass. The mean infarct size was 79 ± 3% of the area-at-risk and 31 ± 3% of the LV mass (raw data not shown).

#### 3.3.2. Cardiac Structure and Function

**Figure 3:**
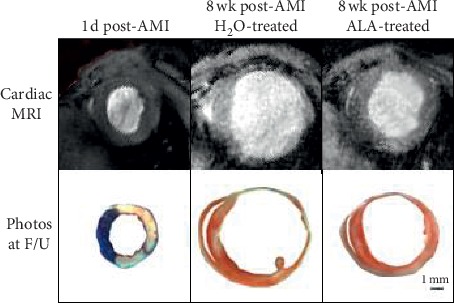
Short-axis cross sections of hearts from control and ALA-treated mice assessed by cardiac MRI and at necropsy. The top panel illustrates typical mid-ventricular, short-axis cardiac MR images from control mice 1 d post-AMI, control mice 8 wk post-AMI and ALA-treated mice 8 wk post-AMI. The bottom panel shows photographs of the corresponding short-axis tissue sections. The pronounced infarct expansion observed in control mice (center panel) was far less evident in mice treated with ALA (panel at right).

**Figure 4:**
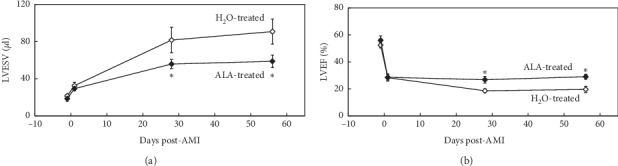
(a) Left ventricular remodeling (LVR) as assessed by cardiac MRI. Daily oral administration of ALA initiated 1 day post-AMI significantly reduced left ventricular end-systolic volume (LVESV) relative to controls, both at 28 and 56 days post-AMI (*P* < 0.05 vs. control at either time point). (b) Left ventricular function as assessed by cardiac MRI. Daily oral administration of ALA initiated 1 d post-AMI significantly preserved left ventricular ejection fraction (LVEF) relative to controls, both at day 28 and 56 days post-AMI (^*∗*^: *P* < 0.05 vs. H_2_O-treated group at either time point).

Heart rates during AMI and subsequent remodeling were comparable between control and ALA-treated mice (Table 1). Mice underwent MRI exams to assess cardiac structure and function 1 day before and 1 day, 28 days, and 56 days after 2-hour LCA occlusion (Figure 3). Both groups were treated identically until 1 day after AMI, and thus no differences in LVESV or LVEF were found between the two groups at baseline or at 1 day post-AMI. When assessed 1 day after reperfusion, AMI had caused a 220% increase in LVESV (from 19 ± 4 *μ*l to 42 ± 2 *μ*l, Figure 4(a)) and a precipitous 55% reduction in LVEF (from 56 ± 5% down to 24 ± 4%, Figure 4(b)). ALA treatment was initiated on day 1 post-AMI. By day 28, mean LVESV in control mice had gradually increased to 79 ± 8 *μ*l (over 4-fold greater than baseline), whereas LVESV in ALA-treated mice (54 ± 7 *μ*l) was significantly smaller (*P* < 0.05). By day 28, LVEF in control mice (21 ± 2%) was essentially unchanged from its value on day 1 post-AMI (19 ± 4%), whereas LVEF in ALA-treated mice had significantly improved to 31 ± 3%. Thus indices of both cardiac structure (LVESV) and function (LVEF) were significantly improved on day 28 post-MI in the ALA-treated group versus control. The cardioprotective effects of ALA treatment were still evident on day 56 post-MI, with no significant changes in either LVESV or LVEF between days 28 and 56 post-AMI in either group (Figure 4). Baseline LVEDV was 45 ± 2 *μ*l, and changes in LVEDV subsequent to MI paralleled those reported for LVESV (data not shown).

**Figure 5:**
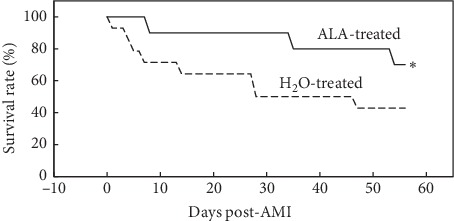
ALA enhanced survival after AMI. By day 28 post-AMI, 9/10 of the mice in the ALA-treated group survived, whereas only 7/14 of the control mice survived. By the end of the study, survival in ALA-treated mice (7/10) was significantly higher in ALA-treated vs. H_2_O-treated mice (6/14). ^*∗*^*P* < 0.05 as compared with the H_2_O-treated group.

Morphometric analysis of the mice at the terminal follow-up on day 56 confirmed that the extent of infarct expansion was better confined, and LV volumes were significantly reduced in ALA-treated versus control mice (Table 1). Importantly, the survival rate on day 28 post-MI in the ALA-treated group (90%) was significantly better than in the control group (50%, *P* < 0.05, Figure 5). The statistical difference in survival rate was maintained through the end of the study (day 56 post-MI), when survival in the ALA-treated group was 70%, whereas it had fallen to 43% in the control group (*P* < 0.05, Figure 5).

#### 3.3.3. Lipid Peroxidation

**Figure 6:**
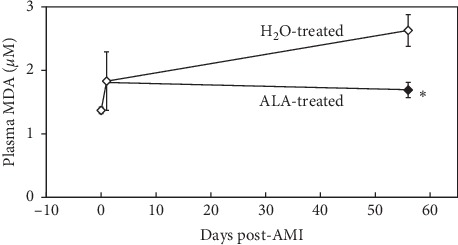
Plasma malondialdehyde (MDA) levels in post-AMI LVR. Plasma MDA was measured on day 1 and day 56 post-AMI. Plasma MDA levels were reduced by 36% in ALA-treated mice versus H_2_O-treated controls, indicating that daily administration of ALA can reduce systemic oxidative stress long after the index event of AMI. ^*∗*^*P* < 0.05 as compared with H_2_O-treated group.

The mean MDA level in ALA-treated mice at day 56 post-MI was reduced by 36% relative to untreated controls (1.7 ± 0.1 vs. 2.6 ± 0.3 *μ*M, respectively, *P* < 0.05, Figure 6). Thus, the increased levels of lipid peroxidation detected late after AMI in control mice were reduced by daily treatment with ALA.

## 4. Discussion

The importance of oxidative stress in the setting of myocardial ischemia/reperfusion injury has long been recognized [1–3]. The enhanced production of ROS as a result of ischemia/reperfusion injury has been detected directly [17, 20] and indirectly [3]. In the current study, a mouse model of AMI was used to demonstrate that oxidative stress exists not only acutely after AMI but also later in the chronic setting of LVR following AMI. Furthermore, we demonstrate for the first time that oral administration of antioxidant therapy not only limits the size of AMI but also preserves cardiac structure and function during LVR post-AMI. The demonstration that ALA is cardioprotective in the chronic setting of LVR after AMI provides additional support for the growing body of evidence indicating that ROS plays an important role in the pathophysiology of LVR and heart failure after AMI [1, 3, 21].

During ischemia, the production of ROS from dysfunctional mitochondria may overwhelm endogenous antioxidant mechanisms [22]. Upon reperfusion, multiple free radical-generating systems, mostly likely NADH oxidase, contribute to a burst of ROS [3, 6, 13, 17]. In the chronic setting of LVR, major sources of ROS may include NAD(P)H oxidases, neutrophils, and even nitric oxide synthase [6, 14, 21, 23]. Chronic production of superoxide curtails the bioavailability of nitric oxide through the formation of peroxynitrite; which in turn leads to vascular dysfunction, contractile dysfunction, activation of the renin-angiotensin II system, ventricular remodeling, and the eventual development of heart failure [24, 25].

In the current investigation, the duration of the coronary occlusion was adjusted to meet the individual requirements of each study (AMI or LVR). In the AMI study, a 45-minute coronary occlusion was chosen so that approximately half of the area-at-risk would be salvaged upon reperfusion, providing maximal sensitivity for the effects of ALA on infarct size. In contrast, a 2-hour occlusion was chosen for the LVR study to produce maximum necrosis and thus induce as much LVR as possible in the weeks that followed [26]. In most previous studies, permanent ligation of the LCA was used to induce LVR [20, 27, 28]. However, the progression of LVR is known to differ between reperfused and non-reperfused myocardium. A reperfused model was chosen for the current study to more closely approximate the prevailing clinical situation in which the myocardium is ultimately reperfused [26].

In the AMI study, mice were pretreated with ALA or vehicle for 1 week before receiving 45 min of LCA occlusion and 24 hours of reperfusion. Mean infarct size expressed as percent of the area-at-risk was reduced by >30% in ALA-treated versus control mice (*P* < 0.05). The increase in lipid peroxidation resulting from AMI was also significantly suppressed in mice pretreated with ALA. Although two doses of ALA were examined (15 and 75 mg/kg·day), there were no statistical differences between these two doses with respect to infarct size or lipid peroxidation (Figures 1 and 2(a)). The plasma level of nitrate/nitrite was also increased in control mice (Figure 2(b)). One of the sources of these increases in lipid peroxidation and nitrite/nitrate was likely from neutrophils infiltrating the ischemic area of the heart as defined by tissue myeloperoxidase activity (Figure 2(c)). This result further confirmed that ALA is a potent antioxidant and plays important role in reducing AMI by inhibiting inflammatory responses. These results are consistent with previous results from another group that demonstrate the infarct-limiting effect of ALA [14].

In the LVR study, treatment with ALA was delayed until 24 hours after the 2-hour coronary occlusion to avoid any possible influence on infarct size. Before initiating treatment, cardiac MRI was used to confirm that AMI had significantly reduced LVEF and increased LVESV to a similar extent in both groups. Subsequent LVR was significantly inhibited in ALA-treated mice as compared with control mice at both 28 and 56 days post-AMI. Similarly, LVEF in ALA-treated mice was significantly improved compared with control mice at both time points (Figure 4). Mean plasma MDA levels in control mice were significantly elevated on day 56 post-AMI, indicating that oxidative stress is present in the myocardium long after AMI. Plasma MDA levels were reduced by 36% in ALA-treated mice versus controls (Figure 6). This study clearly demonstrates that post-AMI LVR is associated with enhanced oxidative stress. Daily oral ALA significantly dampens this oxidative stress, improves LVR, and enhances survival (Figure 5).

When considered in light of the AMI study, our results indicate that oxidative stress exists both early and late after AMI, and daily oral ingestion of ALA significantly reduces oxidative stress in both settings. The cumulative effect of administering ALA before, during, and after AMI was not directly addressed in the current study. However, it is clear that the beneficial effects of such treatment on LVR would be greater than those demonstrated herein since infarct size is the primary determinant of LVR.

### 4.1. Limitations of the Study

Overall, the current study is descriptive in demonstrating that chronic treatment with ALA will increase the resistance of the mouse heart to both AMI and LVR. The results of assays for plasma MDA, NOx and MPO indicate that these pro-oxidative and proinflammatory markers are normalized by chronic ALA treatment, but they do not establish causality. By virtue of its antioxidant and anti-inflammatory properties, it is possible that ALA treatment may also promote endogenous proregenerative processes in the heart after AMI, although this intriguing possibility was not directly investigated in the current study. However, a recent study has shown that ALA may activate cellular survival pathways during AMI [17], which deserves to further investigation in the setting of post-AMI LVR.

## 5. Conclusions

In conclusion, the present study demonstrates the presence of oxidative stress both in the acute phase immediately after AMI and in the chronic remodeling phase following AMI. Daily treatment with ALA not only reduced oxidative stress in both of these settings but also protected the heart against AMI and helped preserve cardiac function and structure during the progression of LVR post-AMI. It should be noted that one of the potential mechanisms of action for ACE inhibitors is through the inhibition of vascular NADH oxidases: potent sources of superoxide and related ROS [21]. Clearly, additional work will be required to determine whether potent antioxidants offer any additional benefit when applied in combination with conventional pharmaceuticals against LVR in the chronic setting after AMI.

## Figures and Tables

**Table 1:** Heart rates and anatomic features during 8-week follow-up in LVR study.

Group	Heart rate (bpm)				Cardiac morphometric data at day 56		
	Baseline	Day 1	Day 28	Day 56	LV vol (*μ*m^3^)^*∗*^	Ht/B Wt^†^	IF extent^‡^
H_2_O	326 ± 21	462 ± 66	380 ± 38	347 ± 17	130.6 ± 33.4	0.67 ± 0.05	40.9 ± 3.7
ALA	360 ± 28	489 ± 47	318 ± 38	358 ± 17	85.6 ± 10.7	0.61 ± 0.03	32.9 ± 2.2
*P* value	NS	NS	NS	NS	<0.05	NS	<0.001

^*∗*^Left ventricular volume; ^†^heart-to-body weight ratio (%); ^‡^infarct extent (%).

## Data Availability

The majority of the data used to support the findings of this study are included within the article. Any additional data (beyond that reported here) are available from the corresponding author upon request.
